# Metabolic investigation in patients with nephrolithiasis

**DOI:** 10.1590/S1679-45082017AO4029

**Published:** 2017

**Authors:** Francilayne Moretto dos Santos, Aline Krampe Peres, Michel Roberto Mandotti, Luis Alberto Batista Peres

**Affiliations:** 1 Centro Universitário Faculdade Assis Gurgacz, Cascavel, PR, Brazil

**Keywords:** Nephrolithiasis/metabolism, Hypercalciuria, Uric acid, Nefrolitíase/metabolismo, Hipercalciúria, Ácido úrico

## Abstract

**Objective:**

To evaluate the prevalence of metabolic disorders associated with nephrolithiasis in a female population.

**Methods:**

A retrospective study on 1,737 patients with evidence of recent formation of renal stones, being 54% females. The laboratory investigation consisted of at least two samples of blood and 24-hour urine to assess calcium, uric acid, citrate and creatinine levels, qualitative cystinuria, urinary pH following fasting and 12-hour water restriction, urine culture, serum creatinine and parathyroid hormone.

**Results:**

The most frequent alterations were hypercalciuria (40.9%), urinary tract infection (23.2%), hypocitraturia (22.4%), low urinary volume (20.5%) and hyperuricosuria (16%).

**Conclusion:**

The most frequent metabolic alterations in females were hypocitraturia, urinary tract infection, low urinary volume and hyperuricosuria.

## RESUMO

**Objetivo:**

Avaliar a prevalência dos distúrbios metabólicos associados à nefrolitíase em uma população feminina.

**Métodos:**

Foi realizado um estudo retrospectivo em 1.737 pacientes com evidência de formação recente de cálculos renais, sendo 54% do sexo feminino. A avaliação laboratorial constou de duas ou mais amostras de sangue e urina de 24 horas com dosagens de cálcio, ácido úrico, citrato e creatinina cistinúria qualitativa, pH urinário em jejum e restrição hídrica de 12 horas, urocultura, creatinina e paratormônio séricos.

**Resultados:**

As alterações mais encontradas foram hipercalciúria (40,9%), infecção do trato urinário (23,2%), hipocitratúria (22,4%), baixo volume urinário (20,5%) e hiperuricosúria (16%).

**Conclusão:**

As alterações metabólicas mais frequentes na população feminina foram hipocitratúria, infecção do trato urinário, baixo volume urinário e hiperuricosúria.

## INTRODUCTION

Nephrolithiasis affects 5-10% of the world population,^(^ ^1^ ^)^ and is more frequent in men than in women - 13 and 7%, respectively.^(^ ^2^ ^)^ Up to 15% of world population will experience one episode of renal calculus during their lifetime, and more than 50% will have a relapse within 10 years.^(^ ^2^ ^,^ ^3^ ^)^ In females, the peak of occurrence is at approximately 30 years of age, and decreases after 50 years, whereas in men, the peak occurs between the fourth and sixth decade of life.^(^ ^3^ ^)^

Kidney stones develop primarily in the renal calyces and pelvis. Their formation is influenced by urinary pH, decreased urinary volume, and presence of bacteria, and the main determinant is urinary supersaturation of crystals. Roughly 70% of calculi are formed by calcium oxalate salts; 15% are composed of magnesium ammonium phosphate (struvite calculi); 5-10% are of uric acid; and 1 to 5%, of cystine.^(^ ^4^ ^)^ When the stones increase in size, they progress with serious damage to the kidney, even with no clinical evidence, and can present complications, such as urinary tract obstruction and infection.^(^ ^5^ ^)^

Several factors are related to predisposition for kidney stones, such as age, sex, sedentary lifestyle, comorbidities (hypertension and diabetes), medications on use, past family or personal history of lithiasis, occupation, diet issues, geographic and climatic aspects, anatomic and metabolic abnormalities.^(^ ^5^ ^)^ The literature has shown a greater incidence of nephrolithiasis in males; however, recent investigations have pointed to an increased incidence in females.^(^ ^6^ ^)^

Major anatomical disorders occur in up to 40% of nephrolithiasis patients, including obstruction of the ureteropelvic junction, horseshoe kidney, complete or incomplete ureteral duplication, medullary sponge kidney, and pelvic kidney.^(^ ^7^ ^)^

## OBJECTIVE

To evaluate the prevalence of metabolic disorders associated with nephrolithiasis in a female population.

## METHODS

This is a retrospective study based on the analysis of medical records of 1,737 adult patients with lithiasis, in the city of Cascavel, Paraná, who were seen at a private nephrology clinic, from 2005 to 2016. Inclusion criteria were spontaneous, endoscopic, and surgical elimination of kidney stones and/or radiologic confirmation of their presence in the urinary tract. The following data were collected: identification, sex, age, family history, clinical presentation, laboratory tests, and imaging tests. The study protocol was approved by the Ethics in Research with Human Beings Committee of the organization, under number 1.604.236, CAAE: 57213316.4.0000.5219.

The laboratory investigation included two or more 24-hour urine samples to evaluate calcium, uric acid, citrate, creatinine, and oxalate; serum calcium, uric acid, creatinine, and parathyroid hormone; urine culture and urine collected after fasting and water restriction for 12 hours, with the objective of researching qualitative cystinuria, crystals in urinary sediment, and urinary pH.

The laboratorial methods used and the reference values adopted for 24-hour urine samples were: for calcium, atomic absorption spectrometry (<4.0mg/kg/day); for uric acid, the enzyme method uricase (<750mg for women and <800mg for men); for citrate, citrate lyase (>320mg); for oxalate, high performance liquid chromatography (>44mg); for creatinine, alkaline picrate (>1,000mg); and for urine volume, the volumetric urine measurement in a Becker collector for visual analysis. For plasma dosing, the methods used were, for calcium, the colorimetric method (8.5 to 10.5mg/dL); for uric acid, uricase colorimetric method (2.0 to 7.0mg/dL); for creatinine, alkaline picrate (0.7 to 1.4mg/dL); and for parathyroid hormone, the intact molecule test (10 to 70ng/L). For tests in isolated urine samples, the methods used were, for qualitative cystinuria, sodium nitroprusside; and for urine pH, measurement by means of reactive strips with an indicator system of pH with methyl red and bromothymol blue. The decreased volume of urine was considered when at least one of the samples showed a 24-hour urine volume lower than 15mL/kg.

Imaging investigation included echography of the urinary tract and a routine excretory urography, as well as computed tomography in selected cases.

For data analysis, the mean, standard deviation, and percentages were calculated. The χ^2^ test and Fisher's exact test were used, as appropriate. The main metabolic disorders were compared between the sexes, as well as the anatomic disorders. For all analyses, p<0.05 was adopted as significant. The statistical treatment of all information was done using the Statistical Package of the Social Science (SPSS) version 15.0 and Excel.

## RESULTS

The medical records of 1,737 patients were examined −952 women (54.8%) and 785 men (45.1%). The male/female ratio was 1:1.2. The symptom most frequently found upon diagnosis was nephritic colic, with a total of 53.3% and 57.4% in women and men, respectively. The family history of lithiasis was positive for 39.8% of women and 40.5% of men.

Out of 902 patients completed the metabolic study, 482 were women (53.4%) and 420 men (46.6%). The most frequent metabolic disorders were hypercalciuria (45.4%), hyperuricosuria (25.1%), hypocitraturia (21.4%), low urinary volume (18%), urinary tract infection (11.6%), hyperoxaluria (5.3%), hyperparathyroidism (4.3%), cystinuria (0.5%), and tubular acidosis (0.4%). When observed by sex, the most common findings in women were hypercalciuria (40.9%), urinary tract infection (23.2%), hypocitraturia (22.4%), low urinary volume (20.5%), and hyperuricosuria (16%). The most common metabolic disorders in males were hypercalciuria (50%), hyperuricosuria (34.3%), hypocitraturia (20.5%), and low urinary volume (15.5%). Table 1 shows all metabolic disorders found.

**Table 1:** Disorders found in 902 patients with nephrolithiasis, according to sex

Metabolic disorders	Female (n=482) n (%)	Male (n=420) n (%)	p value
Hypercalciuria	197 (40.9)	210 (50.0)	0.0059 *
Hypocytraturia	108 (22.4)	86 (20.5)	0.4815 *
Hyperuricosuria	77 (16.0)	144 (34.3)	0.0000 *
Low urinary volume	99 (20.5)	65 (15.5)	0.0492 *
Hyperoxaluria	26 (5.4)	22 (5.2)	0.9170 *
Urinary tract infection	112 (23.2)	13 (3.1)	0.0000 *
Hyperparathyroidism	22 (4.6)	17 (4.0)	0.7035 *
Renal tubular acidosis	1 (0.2)	3 (0.7)	0.3433 †
Cystinuria	3 (0.6)	2 (0.5)	1.0000 †
Undetected disorders	173 (35.9)	47 (11.2)	0.0000 *

* χ^2^

† Fisher's exact test.

The primary anatomic disorders noted were renal cyst, duplex collecting system, obstruction of the ureteropelvic junction, horseshoe kidney, medullary sponge kidney, pelvic kidney, and renal ptosis. Table 2 shows all the anatomic disorders found.

**Table 2:** Anatomic disorders found in 148 patients with nephrolithiasis, according to sex

Anatomical disorders	Female (n=87) n (%)	Male (n=61) n (%)	p value
Renal cyst	32 (36.8)	26 (42.6)	0.4736 *
Duplex collecting system	18 (20.7)	11 (18.0)	0.6885 *
Ureteropelvic junction stenosis	7 (8.0)	5 (8.2)	0.9736 *
Single kidney	6 (6.9)	1 (1.6)	0.2401 †
Atrophic kidney	6 (6.9)	3 (4.9)	0.7367 †
Renal ptosis	3 (3.4)	0 (0)	0.2682 †
Polycystic kidneys	3 (3.4)	3 (4.9)	0.6907 †
Medullary sponge kidney	2 (2.3)	2 (3.3)	1.0000 †
Other disorders	10 (11.5)	10 (16.4)	0.3908 *

* χ^2^;

† Fisher's exact test

## DISCUSSION

The peak incidence of nephrolithiasis occurs between 40 and 60 years of age.^(^ ^3^ ^)^ In this study, we noted that the mean age of patients at the time of metabolic investigation was 49 years, with a slight predominance of women, which could suggest greater compliance of women with the metabolic investigation.

Compatible with the literature, the positive familial history for nephrolithiasis, which varies between 25% and 55%, in this study was about 40%. Family history does not imply merely genetic heritage, since individuals from the same family can share lifestyle habits, such as diet. For example, an elevated intake of sodium has a high impact on the development of hypercalciuria.^(^ ^8^ ^–^ ^10^ ^)^

Distinct metabolic disorders were found in both sexes; while in females there was more urinary infection, in males we saw more hypercalciuria and hyperuricosuria.

Urinary citrate promotes a reduction in the formation and recurrence of kidney stones, since it binds to calcium, inhibiting the spontaneous nucleation and aggregation of oxalate crystals, besides interacting with the Tamm-Horsfall protein to inhibit crystallization of calcium oxalate.^(^ ^2^ ^)^ In the literature, women show more excretion of citrate in urine than men, which decreases the formation of urinary stones in the general female population.^(^ ^11^ ^)^ Some hypotheses to explain this fact include changes in dietary habits, high levels of estrogen, pregnancy, acid-base imbalance, vitamin D and parathyroid hormone.^(^ ^12^ ^)^ In our study there was a higher incidence of hypocitraturia in women with nephrolithiasis submitted to metabolic investigation.

Montilla et al., in a cross-sectional study with 154 women, observed that women showed a protein intake higher than their nutritional needs.^(^ ^13^ ^)^ Considering that the high ingestion of animal-derived proteins has an elevated acid load, urinary citrate decreases due to increased proximal tubular reabsorption and reduced excretion by the tubular cells.^(^ ^12^ ^,^ ^14^ ^)^

In an investigation in North America evaluating the risk of incidence of renal lithiasis in 91,731 healthy women in menopause and post-menopause, it was noted that after a daily administration of conjugated oral estrogen (1.25mg) during 4 days, maintaining a diet restricted to purines, urinary uric acid excretion increased by 23% (p<0.05) in comparison with the baseline value in seven postmenopausal women. Similar results were observed in a study with 22 adult transsexual men treated with estrogen, in which the mean excretion of uric acid increased 28%, with a statistically significant difference.^(^ ^11^ ^,^ ^15^ ^,^ ^16^ ^)^

During pregnancy, we noted innumerous prolithogenic transformations. One of them is physiological hydronephrosis, which leads to urinary stasis, acting as a significant risk factor for the occurrence of kidney stones and urinary infections. Additionally, there is also an increase in the glomerular filtration rate, superior to 40%. Consequently, some lithogenic factors, such as hypercalciuria, elevation of the urinary pH, and intestinal hyperabsorption of calcium undergo an increment in the pregnant woman, favoring supersaturation of salts in urine. On the other hand, the occurrence in pregnancy of some inhibitory factors that reduce the aggregation of crystals and consequently, the formation of urinary stones had already been observed. Among them, we can mention uromodulins, nephrocalcin, glycoproteins, increased urinary pH, and the increase of renal excretion of calcium and magnesium.^(^ ^17^ ^)^

Primary hyperparathyroidism is more common in women.^(^ ^18^ ^,^ ^19^ ^)^ In this disorder, there is an increased release of parathyroid hormone stimulating the synthesis of calcitriol; consequently, the intestinal absorption of calcium and bone reabsorption increase leading to hypercalciuria. This hormone imbalance, along with the rise in vitamin D levels, is one of the causes of nephrolithiasis by increased serum and urine calcium levels.^(^ ^12^ ^)^

In a prior study carried out in Brazil, the metabolic disorders most commonly found were hypercalciuria in 51.8% of patients; hyperexcretion of uric acid in 27.6%; hypocitraturia in 23.5%; low urinary volume in 19.8%; urinary tract infection in 13.5%; hyperoxaluria in 8.0%; hyperparathyroidism in 5.6%; renal tubular acidosis in 0.7%; and cystinuria in 0.7%. Comparing the most prevalent metabolic disorders as per sex, hyperexcretion of uric acid was more frequent in males, and urinary tract infection in females. These data are in agreement with that observed in our study.^(^ ^20^ ^)^

In an investigation performed with 182 patients aged over 12 years, the disorders found, by order of presentation, were hypercalciuria (74%), hypocitraturia (37.3%), hyperoxaluria (24.1%), hypomagnesemia (21%), hyperuricosuria (20.2%), primary hyperparathyroidism (1.8%), secondary hyperparathyroidism (0.6%), and renal tubular acidosis (0.6).^(^ ^21^ ^)^ In our study we noted a lower incidence of hypercalciuria, hypocitraturia and hyperoxaluria, but hyperexcretion of uric acid was greater, and we diagnosed a few cases of cystinuria.

In conformity with literature data, the anatomical alterations most often found were renal cyst, duplex collecting system, and ureteropelvic junction stenosis, in both sexes.^(^ ^7^ ^)^

The treatment strategies for recurrence of renal stones may reduce other comorbidities, as well as the cardiovascular risk in this population, since the association between renal lithiasis and metabolic syndrome was demonstrated. This is a complex situation that encompasses obesity, dyslipidemia, hypertension, *diabetes mellitus,* and insulin resistance.^(^ ^22^ ^)^ In the 4^th^ National Health Inquiry of Portugal, a total of 23,349 questionnaires were applied in individuals aged over 15 years age. There was a significant increase in prevalence of some chronic diseases associated with a greater risk for cardiovascular events in patients with nephrolithiasis relative to the general population, including hypertension (50.4% *versus* 28.6%), obesity (22.7% *versus* 16.9%), *diabetes mellitus* (16.6% *versus* 9.4%), myocardial infarct (3.3% *versus* 1.8%), and stroke (3.8% *versus* 2.1%).^(^ ^23^ ^)^

In addition to reducing comorbidities and recurrences, prevention and treatment of urinary lithiasis results in lower impact in public health, since nephrolithiasis affects primarily adults during their productive age, between the second and the sixth decades of life, and is responsible for a high number of hospital admissions per year, with an elevated cost for the healthcare budget.^(^ ^24^ ^,^ ^25^ ^)^

The limitations of the present study include the fact it is retrospective; moreover, there are limitations inherent to clinical studies, but the data were collected by two investigators, and were homogeneous and trustworthy. The current outpatient clinic was established by the investigator, with the protocol adapted from organizations of excellence in metabolic investigation of nephrolithiasis. The patients analyzed were evaluated by other specialties and referred to nephrology, specifically for the metabolic investigation of lithiasis. As to urinary infection, the patients were referred with a history of repeated urinary tract infections, with posterior formation of stones, and were not yet treated by the investigator at the time of the infection. Urinary sodium was not routinely dosed; nevertheless, all patients had calciuria established. In hypercalciuria patients, dietary sodium restriction was recommended. Natriuria could be used to evaluate sodium intake, and is important in assessing compliance to diet. There are reports in literature with no assessment of urinary sodium.^(^ ^26^ ^)^ Urinary potassium and magnesium were not dosed either, and citraturia was used to evaluate crystallization inhibitors.

Future studies are needed to better understand the metabolic disorders in female patients, decrease the recurrence of nephrolithiasis, and consequently, reduce the cardiovascular risk in this population.

## CONCLUSION

The most frequent metabolic disorders in the female population were hypocitraturia, urinary tract infection, low urinary volume, and hyperuricosuria. Distinct metabolic disorders were found in both sexes – women presented more cases of urinary infection, and men, hypercalciuria and hyperuricosuria.
